# Pioglitazone alters monocyte populations and stimulates recent thymic emigrants in the BBDZR/Wor type 2 diabetes rat model

**DOI:** 10.1186/s13098-015-0068-6

**Published:** 2015-09-02

**Authors:** Bradley T. Gao, Ryan P. Lee, Youde Jiang, Jena J. Steinle, Vanessa M. Morales-Tirado

**Affiliations:** Department of Ophthalmology, College of Medicine, The University of Tennessee Health Science Center, Memphis, TN 38163 USA; Department of Anatomy and Neurobiology, College of Medicine, The University of Tennessee Health Science Center, Memphis, TN 38163 USA; Department of Microbiology, Immunology and Biochemistry, College of Medicine, The University of Tennessee Health Science Center, Memphis, TN 38163 USA; Department of Anatomy and Cell Biology, Wayne State University School of Medicine, Detroit, MI USA

**Keywords:** Pioglitazone, Type 2 diabetes, Immunomodulation, Recent thymic emigrants

## Abstract

**Background:**

Type 2 diabetes is commonly characterized by insulin deficiency and decreased sensitivity of insulin receptors, leading to a chronic state of hyperglycemia in individuals. Disease progression induces changes in the immune profile that engenders a chronic inflammatory condition. Thiazolidinedione (TDZ) drugs, such as Pioglitazone (Pio), aid in controlling disease symptoms. While the mechanisms by which Pio controls hyperglycemia are beginning to be understood, relatively little is known about the effects of Pio on suppression of the systemic immune phenotype, attributed to visceral adipose tissue and macrophages.

**Methods:**

Here, we utilize the recently developed BBDZR/Wor type 2 diabetes rat model to test our hypothesis that a selective in vivo growth of CD3^+^T cells in the spleen contributes to the increase in T lymphocytes, including Tregs, independent of visceral adipose tissue. We investigated the systemic effects of Pio on multifactorial aspects of the disease-induced immune phenotype both in vivo and in vitro in normal, non-diabetic animals and in disease.

**Results:**

Our work revealed that Pio reversed the lymphopenic status of diabetic rats, in part by an increase in CD3^+^ T lymphocytes and related subsets. Moreover, we found evidence that Pio caused a selective growth of newly differentiated T lymphocytes, based on the presence of recent thymic emigrants in vivo. To investigate effects of Pio on the inflammatory milieu, we examined the production of the signature cytokines TNF-α and IL-1β and found they were reduced by Pio-treatment, while the levels of IL-4, an anti-inflammatory mediator, were significantly increased in a Pio-dependent manner. The increase in IL-4 production, although historically attributed to macrophages from visceral adipose tissue under other conditions, came also from CD3^+^ T lymphocytes from the spleen, suggesting splenocytes contribute to the Pio-induced shift towards an anti-inflammatory phenotype.

**Conclusions:**

We show for the first time that Pio treatment significantly suppresses the systemic inflammatory status in the BBDZR/Wor type 2 diabetes rat model by the selective growth of newly differentiated CD3^+^ T cells and by increasing CD3^+^IL-4 production in immigrant spleen lymphocytes.

## Background

Due to a persistent rise in incidence, type 2 diabetes affects 29.1 million Americans, or 9.3 % of the US population, and the disease ranks as the 7th leading cause of death in 2010 [1–3]. In 2012, diabetes and its complications accounted for over $245 billion in direct medical costs and indirect expenses related to wages and lost work. Type 2 diabetes represents 90 % of the total cases diabetes mellitus nationwide, and current treatment options range from exercise and diet modification to pharmaceutical interventions that augment insulin sensitivity and alleviate hyperglycemia [1]. While many of the pathological consequences of type 2 diabetes are related to chronic hyperglycemia, the onset and progression of this disease also leads to a dysregulation of the immune response, resulting in chronic inflammation [4]. Therefore, in addition to combating the effects of glucose toxicity, effective treatments must concurrently mitigate the activation of inflammatory mediators in order to prevent immune-mediated pathology [4].

Thiazolidinediones (TZDs) are a common class of drugs prescribed to patients with type 2 diabetes. TZDs, such as rosiglitazone and pioglitazone, are used to lower systemic glucose concentration by insulin sensitivity [5, 6]. These compounds function as peroxisome proliferator-activated receptor gamma (PPARγ) agonists [7]. Activation of PPARγ in tandem with the retinoid X receptor modulates regulatory effects on inflammatory target genes [8, 9]. The cumulative result is decreased insulin resistance [10–13], modification of adipocyte differentiation [11, 12, 14, 15], decreased leptin levels [16], and decreased mean arterial pressure [13].

The present study aimed to assess the mechanisms responsible for systemic effects of Pioglitazone (Pio) on the immunoregulation of lymphocytes and monocytes. For these studies, we analyzed secondary immune organs in the recently developed BBZDR/Wor type 2 diabetes rat model, thus allowing us to examine effects of Pio on altered systemic immune function associated with the disease status. Past studies have examined anti-inflammatory mechanisms of PPARγ agonists, such as Pio and Ciglitazone [17–19], and report down-regulation of macrophages [20, 21] and modulation of T cell activation [22–24]. However, most of these studies focus on analyses of the macrophage and regulatory T cell population in adipose tissue and not in immune organs such as the spleen and liver. Here, we demonstrate a selection for in vivo growth of T lymphocytes, indicated by an increase in recent thymic emigrants, as a result of Pio treatment in BBDZR/Wor rats. Furthermore, to investigate extra-splenic contributions, we studied the in vitro effects of Pioglitazone in normal, non-diabetic, splenocytes and demonstrate an increase in T cells, including regulatory T cells, and in monocytes, independent of the presence of visceral adipose tissue.

## Methods

### Animals

Male obese and lean (non-diabetic) littermates BBZDR/Wor rats were purchased from Biomedical Research Models (Worchester, MA). Obese: diabetic model; lean littermates: non-diabetic, control group. Any obese rat that did not show glucose levels >250 mg/dL were excluded from the study. Five rats from each group received a daily intraperitoneal (i.p., 1 mL final volume) injection of 25 mg/kg Pioglitazone or vehicle (5 % DMSO) for 2 months, as described in [19], followed by sacrifice for analyses. All animal experiments were approved by The University of Tennessee Health Science Center Institutional Animal Care and Use Committee.

### Glucose measurements

Rats underwent glucose measurements by glucose strip readings, as we previously described [25]. Rats glucose levels >250 mg/dL were considered diabetic [19].

### Tissue culture experiments

Splenocytes and hepatocytes were isolated by physical disruption using the back end of a syringe on a 70 μM nylon strainer. Cell suspension was washed and Red Blood Cell (RBC) contaminants were eliminated using RBC Lysis Buffer (BioLegend, San Diego, CA, USA) following manufacturer’s instructions. Cells (5.0 × 10^5^ cells/mL) were cultured in a 24-well plate in RPMI/10 % FBS/1 % Pen/Strep media. In vitro conditions included ±25 μM Pioglitazone (Pio; Sigma-Aldrich, St. Louis, MO, USA) for 24 h. Ex vivo cell activation was done using Phorbol 12-myristate 13-acetate (PMA; Sigma-Aldrich) at a concentration of 100 ng/mL for 6 h. Golgi Stop^®^ was added (1 μL per 1 mL of culture) for the last 4 h of incubation prior to harvest to inhibit cytokine transport.

### Surface molecule labeling

Cell suspensions were washed in Staining Buffer (PBS/1 % FBS) prior to labeling. Cells were labeled for 30 min protected from light on ice bucket. Once labeled cells were washed multiple times with Staining Buffer and analyzed immediately. The following anti-rat antibodies were used: anti-CD3 AF-647, anti-CD4 PE-Cy7, anti-CD11b/c APC, anti-CD25 FITC, and anti-CD31 Biotin/Streptavidin Brilliant Violet 421 (SA-bv421), all from BioLegend (San Diego, CA, USA). For each antibody we used the respective Immunoglobulin G (IgG) isotype control. In addition, we used single label control Abc™ Capture Beads (Life Technologies). Data acquired in BD Biosciences LSRII Flow Cytometer and analyses were done using FlowJo vX.10.0.6 software (Tree Star, OR).

### Intracellular assay

Following cell surface labeling, cells were fixed using eBioscience FoxP3 Fixation/Permeabilization Buffer (eBiosciences), followed by intracellular labeling for 30 min with either anti-rat FoxP3 PE or isotype control PE. Cells were washed multiple times prior acquisition and analysis.

### Western blot

Cells were lysed in 25 μL of Lysis Buffer as previously described [26]. Protein concentrations were determined using Pierce™ BCA Protein Assay Kit (Thermo Scientific). A total of 30 μg of denatured protein was used for each sample loaded in a Novex^®^ 4–12 % Tris–Glycine Gel (Life Technologies). Membrane was blocked in 20 mL of 5 % BSA in TBS-Tween 20 and incubated overnight at 4 °C in 1:1000 PPARγ rabbit monoclonal antibody (Cell Signaling Technologies) followed by 1:1000 anti-rabbit secondary antibody solution for 1 h at RT. Once completed, the membrane was washed and probed for β-actin as control (Cell Signaling Technologies). SuperSignal West Pico Chemiluminiscent Substrate (Thermo Scientific) was used. Densitometry analysis was done using Kodak Molecular Imager.

### Statistics

Mann–Whitney U tests were used for Western Blot data and student t tests were used for flow cytometry analysis. Data was analyzed using GraphPad software (GraphPad Software, Inc., La Jolla, CA, USA). Values of *p* < 0.05 were considered significant.

## Results

### Pio eliminates hyperglycemia without a reduction in adipose tissue

Recently, we provided evidence that Pio improved retinal insulin signaling in the BBZDR/Wor type 2 diabetes rat model in a PPARγ-dependent manner [19]. Pio reduced TNF-α and SOCS3-activated insulin resistance pathways in the retina and protected against diabetic retinopathy [19]. As a next step, we wanted to assess the systemic immunomodulatory effects of Pio on BBZDR/Wor rats under the same treatment conditions.

**Fig. 1:**
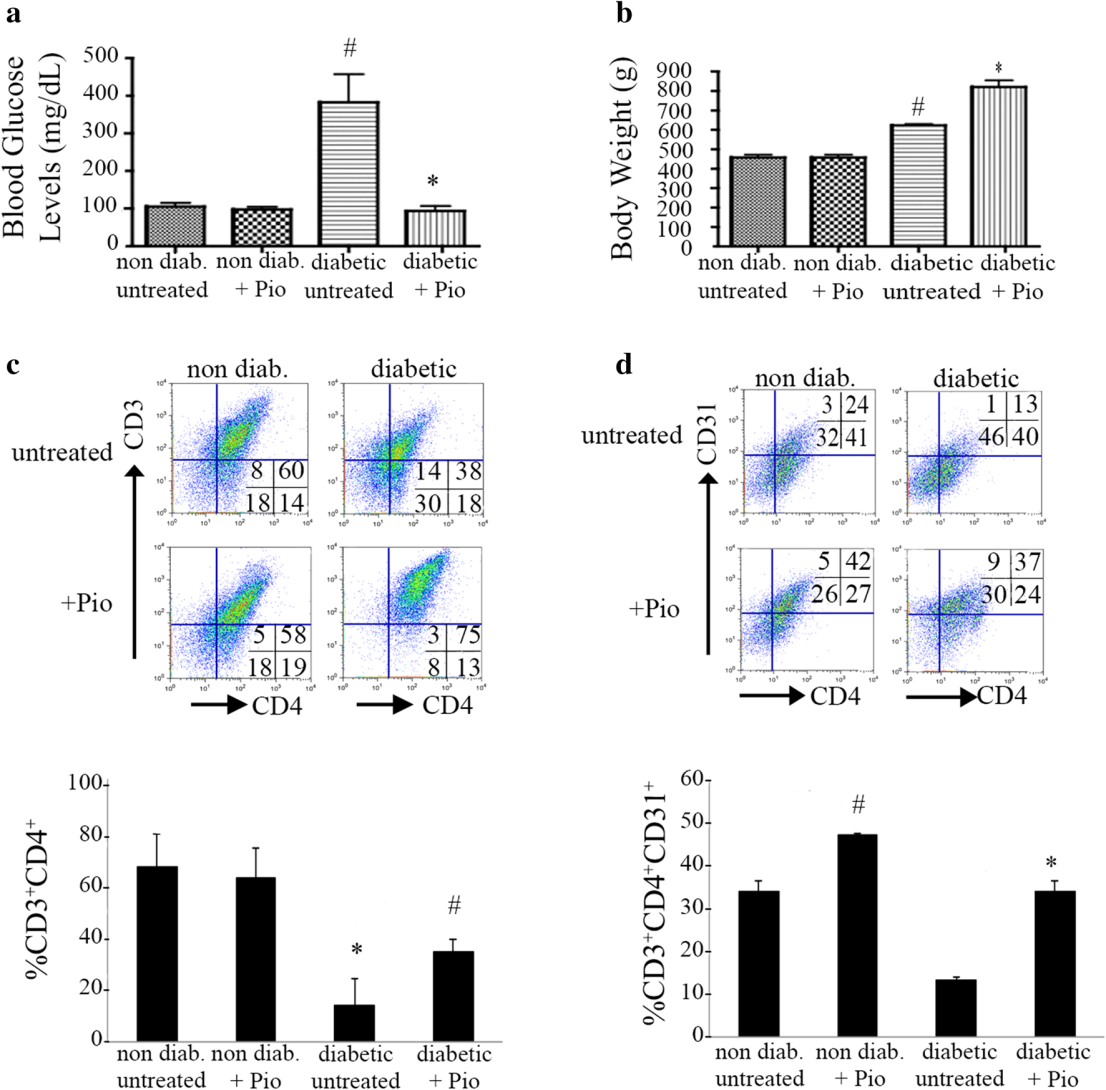
Pioglitazone eliminates hyperglycemia and mitigates lymphopenia in BBZDR/Wor diabetic rats. **a**, **b** Pioglitazone-dependent reduction in glucose levels is independent of weight. BBDZR/Wor diabetic and non-diabetic (littermates) rats were injected daily intraperitoneally with either 25 mg/kg pioglitazone or vehicle (5 % DMSO) for 2 months. Each group had 5 rats (**a** *p < 0.05, ^#^ *p* < 0.0002; **b** ^#^ *p* = 0.0002, *p = 0.0018). **c** Pioglitazone restores diabetic-induced lymphopenic status. Splenocytes from all four groups were examined for the percentage of CD3^+^CD4^+^ T cells using rat anti-CD3 Alexa Fluor 647 and anti-CD4 PE-Cy7 antibodies. *Upper* representative pseudocolor plot of 1 rat per condition. *Lower* quantitation of the flow cytometry analysis (n = 3; *p = 0.0087, ^#^ *p* = 0.0043). **d** In vivo increase in percentage of T lymphocytes is partly attributed to increase in recent thymic emigrants. Gated CD3^+^ T cells were examined for CD4 and CD31 immunoreactivity by using rat anti-CD4 PE-Cy7 and rat anti-CD31 Biotin/Streptavidin bv421, respectively. *Upper* representative plot of 1 rat per condition. *Lower* quantitation analysis (n = 3; ^#^ *p* < 0.005, *p = 0.0011)

First, we confirmed the efficacy of Pio in reducing hyperglycemia in this model. Ten obese diabetic BBZDR/Wor rats and ten lean non-diabetic littermates were randomized into treatment or control, yielding four groups (diabetic, diabetic + Pio, non-diabetic, non-diabetic + Pio). For the five rats in each of the two treatment groups, 25 mg/kg of Pio was injected intraperitoneally each day for 2 months. Figure 1a shows a dramatic reduction in blood glucose levels in the diabetic rats after Pio treatment (*p* < 0.05). Consistent with established mechanisms of Pio action, glucose reduction was not associated with loss of body mass (diabetic versus non diabetic: 383 ± 14.8 versus 105.3 ± 20.8; *p* = 0.0002; diabetic + Pio versus diabetic: 93.4 ± 28.1 versus 383 ± 14.8; *p* = 0.0140). Moreover, Pio-treated rats gained weight compared to untreated diabetic rats (Fig. 1b, diabetic versus non diabetic: 623 ± 12.3 versus 457 ± 29.7; *p* = 0.0002; diabetic + Pio versus diabetic: 820.8 ± 75.9 versus 623 ± 12.3; *p* = 0.0018). These results confirm that the effect of Pio on glucose normalization and the immunophenotype is independent of loss of adipose tissue.

### Pio mitigates lymphopenia in BBZDR/Wor rats

In addition to hyperglycemia and obesity, diabetic BBZDR/Wor rats have reduced numbers of circulating T lymphocytes compared to their non-diabetic lean littermates. Using splenocytes as an indicator of the systemic immunophenotype, we next determined differences in the relative levels of CD3^+^ and CD3^+^CD4^+^ splenocyte subtypes between the groups. As shown in Table 1 and Fig. 1c, the percentage of CD3^+^ and CD3^+^CD4^+^ T cells among splenocytes is lower in the untreated diabetic rats compared to untreated non-diabetic lean controls [CD3^+^: 10.7 % ± 3.0 versus 86.4 % ± 2.8, respectively, *p* = 0.0087; CD3^+^CD4^+^: 11.0 % ± 8.4 versus 53.1 % ± 10, respectively, *p* = 0.0043; (mean ± SD)]. Treatment of non-diabetic rats with Pio had no significant effect on the percentage of CD3^+^ T cells (86.2 % ± 0.5, *p* = 0.4474) and a marginally, but not significant increase on the percentage of CD3^+^CD4^+^ T cells (*p* = 0.05). However, treatment of the diabetic rats with Pio significantly increased the percentage of both CD3^+^ (64.3 % ± 3.3 versus 10.7 % ± 3.0, *p* = 0.0135) and CD3^+^CD4^+^ T cells (27.3 % ± 3.9 versus 11.0 % ± 8.4, *p* = 0.0302). This increase reversed the lymphopenia observed in the diabetic obese rats compared to their non-diabetic littermates.

**Table 1:** Splenocyte immunophenotypes after treatment with Pioglitazone in vitro and in vivo

	In vitro		In vivo			
	Non-diab.	Non-diab. + Pio	Non-diab.	Non-diab. + Pio	Diabetic	Diabetic + Pio
CD3^+^	14.4 % ± 0.4	17.8 % ± 1.0*	86.4 % ± 2.8	86.2 % ± 0.5	10.7 % ± 3.0*	64.3 % ± 3.3*
CD3^+^CD4^+^	12.7 % ± 0.7	3.7 % ± 0.3^#^	53.1 % ± 10	50.0 % ± 8.8	11 % ± 8.4^#^	27.3 % ± 3.9^#^
CD3^+^CD4^+^CD25^+^	6.6 % ± 0.4	33.1 % ± 3.4^¶^	12.4 % ± 1.8	19.0 % ± 0.5	4.6 % ± 2.1^¶^	36.2 % ± 7.1^¶^

Splenocytes were labeled with rat anti-CD3 AF-647, anti-CD4 PE-Cy7, and anti-CD25 FITC. Results are % ± SD, n = 3

In vitro non-diabetic + Pio versus non-diabetic: * *p* = 0.0085, ^#^ *p* = 0.0006, ^¶^ *p* = 0.0022

In vivo diabetic versus non-diabetic (1): * *p* = 0.0087, ^#^ *p* = 0.0043, ^¶^ *p* = 0.0040

Diabetic + Pio versus Diabetic (2): * *p* = 0.0135, ^#^ *p* = 0.0302, ^¶^ *p* = 0.0178

### In vivo increase of T lymphocytes by Pio correlates with increase in recent thymic emigrants

To investigate the origin of this Pio-stimulated population of lymphocytes, we labeled splenocytes in each group with anti-CD31 monoclonal antibody. Positivity of CD31 among CD3^+^CD4^+^ T lymphocytes is a marker of recent thymic emigration (reviewed in [27]). Figure 1d demonstrates a significant increase in the percentage of CD3^+^CD4^+^CD31^+^ splenocytes after Pio treatment in both diabetic (13.2 % ± 1.0 versus 34.2 % ± 2.3, *p* = 0.0011) and non-diabetic rats (34.1 % ± 2.6 versus 47.4 % ± 0.6, *p* < 0.005). These results suggest a Pio-dependent amelioration of lymphopenic status in the BBZDR/Wor diabetic rats due, at least in part, to lymphocyte emigration from the thymus after Pio treatment.

### In vivo decrease in pro-inflammatory cytokines (TNF-a and IL-1β) and increase in anti-inflammatory cytockine, IL-4, after Pio treatment

**Fig. 2:**
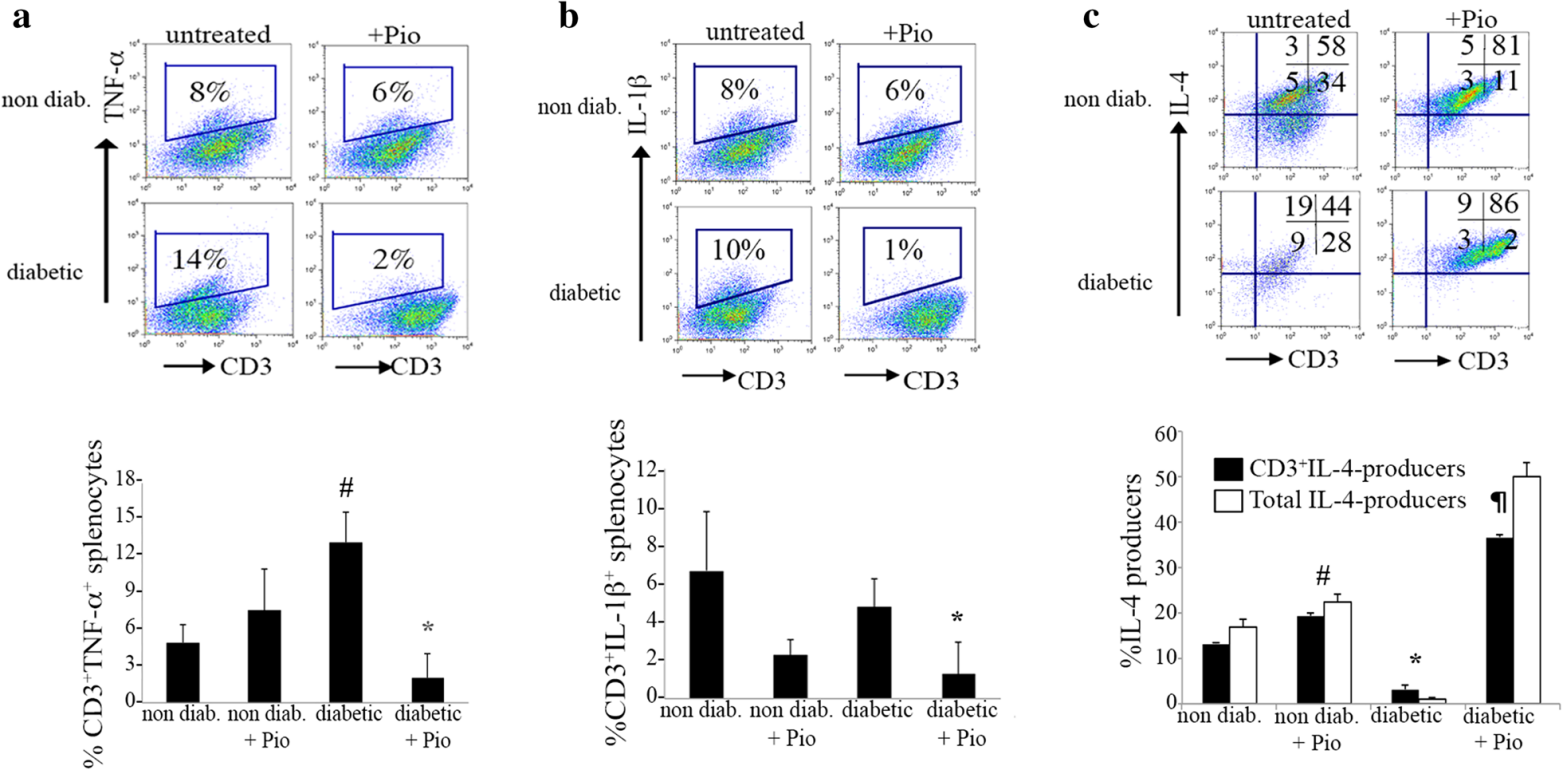
Reduced inflammatory phenotype in pio-treated diabetic BBDZR/Wor rats. Splenocytes from all four groups were cultured for 6 h ex vivo in the presence of Phorbol 12-myristate 13 acetate (PMA) at a concentration of 100 ng/mL. Golgi Plug was added for the last 4 h of treatment to inhibit protein transport. Cells were harvested and analyzed for different cytokines. Gates were established by using respective anti-cytokine isotype control. *Upper panels* are representative from n = 3 and *lower panels* (*bar graphs*) represent % ± SD. **a** Results for CD3^+^TNF-α-producers (^#^ *p* < 0.005, *p* < 0.05); **b** CD3^+^IL-1β-producers (* *p* < 0.05); and **c** CD3^+^IL-4 and total IL-4 producers (^#^ *p* < 0.0005, *p < 0.005, ^¶^ *p* < 0.05)

As has been extensively shown and reviewed [28–34], states of obesity and hyperglycemia upregulate pro-inflammatory cytokines both locally and systemically. To confirm that the diabetic BBZDR/Wor rats exhibit these pathologic changes, we investigated the percentage of CD3^+^ splenocytes producing the pro-inflammatory cytokines TNF-ɑ and IL-1β. As expected, diabetic rats showed an increase in the percentage of CD3^+^TNF-ɑ-producing cells compared to the non-diabetic (11.7 % ± 1.2 versus 4.4 % ± 1.3, *p* < 0.005, Fig. 2a). Furthermore, our data suggests splenic lymphocytes contribute, at least part, to the pro-inflammatory cytokine milieu. Our results suggest that at least part of that action, in the case of Pioglitazone, is due to reduction of the CD3^+^ T cells that produce TNF-α and IL-1β. Diabetic rats treated with Pio had a lower percentage of CD3^+^TNF-α-producers (1.8 % ± 1.7 versus 11.7 % ± 1.2 in controls; *p* < 0.05, Fig. 2a). Similarly, CD3^+^IL-1β-producers were reduced from 5.0 % ± 1.4 to 1.3 % ± 1.6 (*p* < 0.05, Fig. 2b).

Next, we addressed if the reduction in pro-inflammatory mediators was accompanied by an increase in anti-inflammatory cytokines, such as IL-4. First, we show BBZDR/Wor diabetic rats have significantly diminished levels of CD3^+^IL-4^+^ splenocytes compared to non-diabetics (1.1 % ± 0.1 versus 13.5 % ± 1.3, respectively *p* < 0.005, Fig. 2c). As expected, there was a shift towards an anti-inflammatory phenotype with a significant increase in CD3^+^IL-4-producers in the spleen in both the non-diabetic and the diabetic treated groups (non-diabetic: 13.5 % ± 1.3 to 22.2 % ± 1.9, *p* < 0.0005; diabetic: 1.1 % ± 0.1 to 30.4 % ± 15, *p* < 0.05; Fig. 2c). Previous reports showed the source of IL-4 production was from a macrophage population within adipose tissue, termed M2 [35, 36]. To investigate the relative contribution of the CD3^+^ T cell population to the total IL-4 production, we analyze for the total IL-4-producer cells, comprising both CD3^+^ and CD3^neg^ cells. Our results revealed that CD3^+^ T cells are large contributors, in both the non diabetic and diabetic groups, as shown in Fig. 2c. CD3^+^ contribution to the IL-4-producers in the non-diabetic + Pio group was 43.9 versus 80.2 % in the diabetic + Pio group. Together, these results implicate CD3^+^ splenocytes as contributors to the loss of anti-inflammatory signaling associated with diabetes. Furthermore, Pio treatment is able to recover critical regulatory balance in the immunophenotype.

### Pio reduces CD11b/c^+^ population in BBZDR/Wor rats

**Fig. 3:**
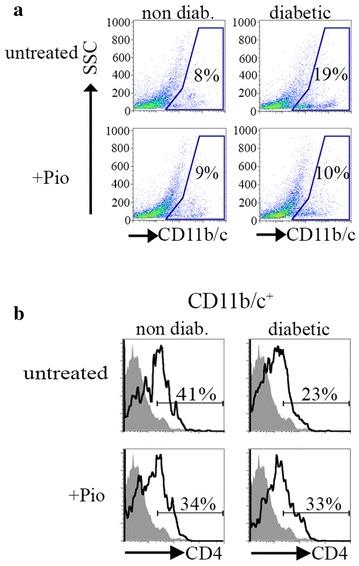
Pioglitazone alters CD11b/c^+^ population in splenocytes of BBZDR/Wor rats. **a** Splenocytes from all groups were labeled with rat anti-CD11b/c APC. **b** Gated population in *panel* **a** examined for expression of CD4. Figures are representative of 2-pooled splenocytes per condition

Recent literature has placed a significant emphasis on macrophages, particularly those in adipose tissue, as drivers of obesity-related inflammation and subsequent insulin resistance [37, 38]. Specifically, there is a decrease in anti-inflammatory M2 macrophages and a reciprocal increase in pro-inflammatory M1 macrophages. These M1 macrophages are defined as CD11c^+^, which have been shown to increase in adipose tissue with obesity [39]. We labeled Pio-treated and untreated splenocytes with an anti-CD11b/c antibody that reacts with a common epitope within the CR3 complement receptor (C3bi) shared by CD11b and CD11c. This epitope is non-exclusive, as it is present in monocytes, dendritic cells, macrophages, and other immune cells. The percentage of splenocytes expressing this marker was higher in diabetic rats compared to non-diabetic littermates (19 versus 8 %; 2.4-fold increase, Fig. 3a). After Pio treatment, the percentage of CD11b/c^+^ splenocytes decreased to levels comparable to non-diabetic controls (diabetic: 10 %, non-diabetic: 9 %). Interestingly, Pio treatment did not significantly affect levels of this marker among treated and untreated non-diabetic rats (9 and 8 %, respectively). Next, we analyzed these CD11b/c^+^ cells by examination of CD4 positivity. Although the percentage of CD11b/c^+^ cells was higher in diabetic rats, a much smaller percentage of these cells were CD4^+^ (23 % in diabetic versus 41 % in non-diabetic, Fig. 3b). Pio treatment partially reversed this imbalance by increasing the percentage of CD4^+^ among the CD11b/c^+^ population in the diabetic rats from 23 to 33 %.

### Reduction of CD4^+^ T lymphocytes, increase in regulatory T cells (CD3^+^CD4^+^CD25^+^FoxP3^+^) and modulation of CD11b/c^+^ cells after Pio treatment in vitro

**Fig. 4:**
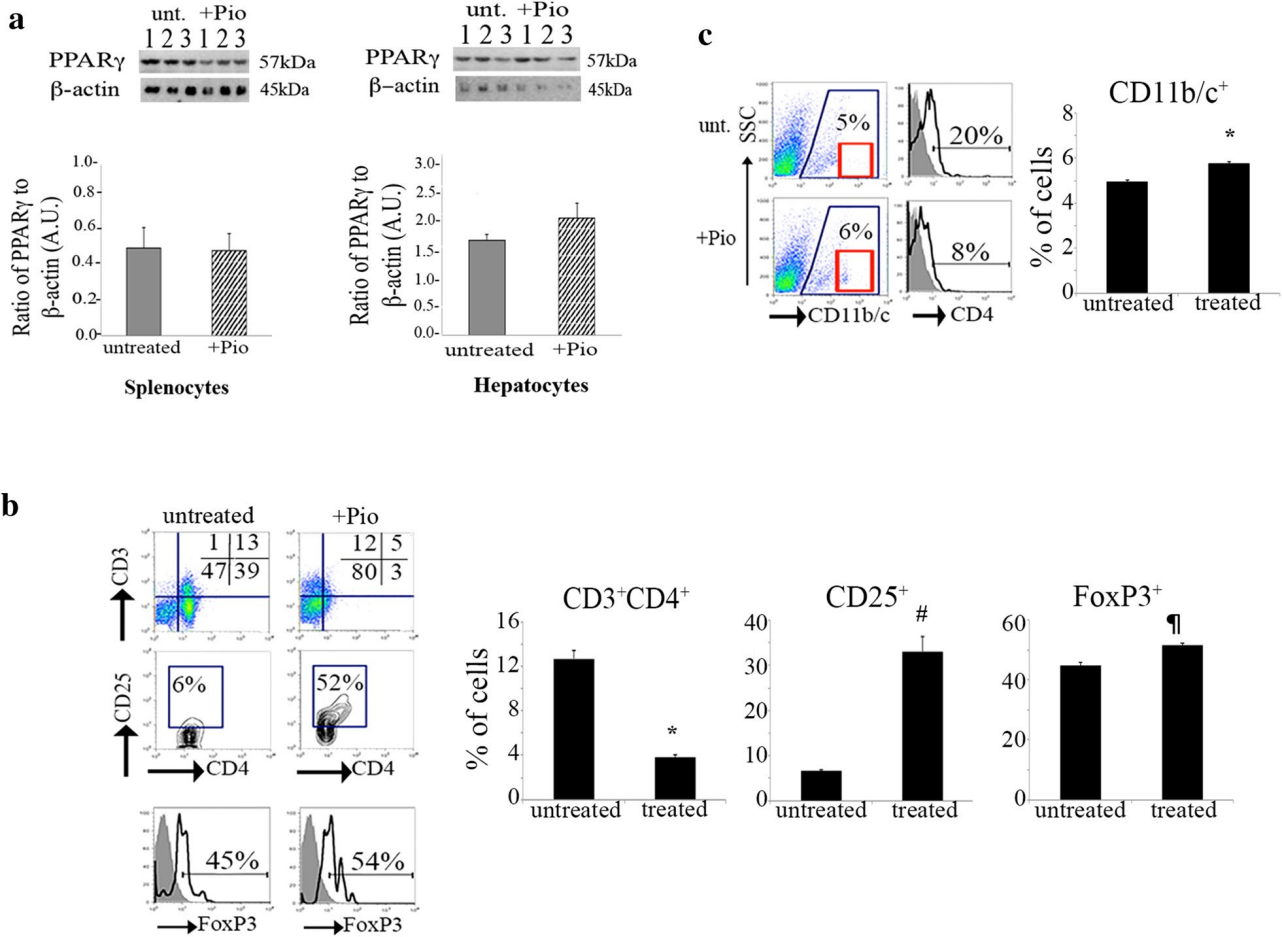
In vitro effects of Pioglitazone treatment in non-diabetic splenocytes. **a** *Left*, Similar levels of PPARγ in splenocytes with and without Pioglitazone treatment. *Right*, Increase in PPARγ levels in Pio-treated hepatocytes compared to untreated. Splenocytes or hepatocytes were cultured in the presence or absence of Pioglitazone 25 μM for 24 h followed by cell lysis treatment for Western blot analysis. Results are shown as *bar graphs*, *dark gray* represent untreated, *white with black stripes* represent Pio-treated. *Graphs* show the ratio of PPARγ to β-actin as arbitrary units (A.U.); n = 3. *Upper part* shows blot results in presence (+Pio) or absence (untreated) of treatment. **b** *Top panels* show the percentage of CD3^+^ and CD3^+^CD4^+^ T lymphocytes in untreated and Pio-treated splenocytes; n = 3. Cultured splenocytes were harvested and surface labeled with anti-CD3 Alexa Fluor 647, -CD4 PE-Cy7 and -CD25 FITC antibodies prior fixation and permeabilization for intracellular FoxP3-PE labeling. *Mid panel* shows percentage of CD3^+^CD4^+^CD25^+^ T cells followed by FoxP3 analysis of the gated population. *Histogram* shows percentage of FoxP3^+^ cells from the CD3^+^CD4^+^CD25^+^ gate; n = 3. *Gray histogram* depicts isotype control; *black line* depicts anti-rat FoxP3 labeling. Figures are representation of n = 3. At the* right* there is quantitation of each *panel* in *bar graph*: *p = 0.0006, ^#^ *p* = 0.002, ^¶^ *p* = 0.0237

After establishing that Pio treatment of BBZDR/Wor diabetic rats mitigates several aspects of the pro-inflammatory immunophenotype, we investigated which specific effects were mediated by extra-splenic mechanisms and which were due to direct effects by splenocytes. Splenocytes from non-diabetic rats were cultured in vitro for 24 h in the presence or absence of 25 μM Pio. Western blot analysis showed no statistical difference (*p* = 0.35; n = 3) in the levels of PPARγ protein relative to β-actin in splenocytes cultured with or without Pio (Fig. 4a, left). For comparison, we cultured hepatocytes from the same rats under the same conditions. Figure 4a, right demonstrates that levels of PPARγ protein levels in hepatocytes treated with Pio although higher, are not significant (*p* = 0.05, n = 3).

Next, we examined the percentage of total CD3^+^ T lymphocytes in splenocytes cultured in the presence of Pio. As we found in vivo, the percentage of CD3^+^ cells present in splenocyte cultures increased after treatment (17.8 % ± 1.0 treated versus 14.4 ± 0.4 control, *p* = 0.0085) (shown in Table 1 and Fig. 4b). Furthermore, there was a prominent down-regulation of the CD4 co-receptor among CD3^+^ cells, as 3.7 % ± 0.3 of Pio-treated splenocytes exhibited a CD3^+^CD4^+^ phenotype compared to 12.4 % ± 0.6 in untreated cells (*p* = 0.0006). These results suggests that the in vivo increase in CD3^+^CD4^+^ cells in the diabetic rat spleens was in large part due to recent thymic emigration after Pio treatment. Additionally, Pio treatment exerted significant effects without upregulating PPARγ protein levels. We further characterized the CD3^+^CD4^+^ subset to evaluate the percentage of CD3^+^CD4^+^ T lymphocytes immunoreactive against CD25. Upregulation of the IL-2Rα chain, CD25, is associated with an activated and immunoregulatory phenotype. We hypothesized that CD25 expression would be increased and partially responsible for the anti-inflammatory transition after Pio treatment. Figure 4b shows the percentage of CD3^+^CD4^+^CD25^+^ T lymphocytes in untreated versus Pio-treated cells. In the untreated samples, an average of 6.6 % ± 0.4 of CD3^+^CD4^+^ cells co-expressed CD25 (Table 1). However, Pio-treated splenocytes showed an increased percentage of CD3^+^CD4^+^CD25^+^ T cells: 33.1 % ± 3.4 (*p* = 0.0022). To further explore this immunomodulation we monitored the percentage of cells that were FoxP3 positive, a well-established marker for immunoregulatory phenotype associated with relative immunosuppression [40–42]. While both groups showed a large population of CD3^+^CD4^+^CD25^+^FoxP3^+^ cells, this subpopulation increased from 44.7 % ± 2.5 to 51.5 % ± 2.1 (*p* = 0.0237) after Pio treatment. Collectively, this data suggests treatment with Pio favors an anti-inflammatory phenotype.

Lastly, we determined the effects of Pio on other immune populations by labeling Pio-treated and untreated splenocytes with anti-CD11b/c as before. Figure 4c shows a significant increase in the percentage of cells with a CD11b/c^+^ phenotype in untreated versus Pio-treated cells (4.92 % ± 0.09 versus 5.73 % ± 0.11, respectively; *p* = 0.0007).

## Discussion

Two decades ago, Lehmann et al. [6], identified thiazolidinedione (TZD) derivatives as potential anti-diabetic agents in animal models of type 2 diabetes. These drugs exhibit high affinity for the peroxisome proliferator-activated receptor gamma (PPARγ) ligand. PPARγ belongs to a family of nuclear receptors that play central roles in the regulation of metabolic homeostasis, including modulation of lipids, glucose, and bone remodeling [43–45]. This class of receptor is considered a regulator of adipose tissue inflammation and its activation increases the expression of adiponectin enhancing insulin sensitivity. Pioglitazone (Pio) is a PPARγ agonist used for the treatment of type 2 diabetes [46, 47]. While Pio has extensively been studied in resident cells within adipose tissue, the effects of this drug are poorly understood in secondary immune organs, such as the spleen and liver. Our work demonstrates that Pio may reverse some of the type 2 diabetes-associated pathogenesis by a de novo increase of CD3^+^CD4^+^CD31^+^ T lymphocytes, with a concomitant reduction of pro-inflammatory mediators (TNF-α, IL-1β) and augmentation of anti-inflammatory cytokines (IL-4) in the BBDZR/Wor type 2 diabetes rat model.

Obesity, implicated in the etiology of type 2 diabetes, causes a chronic low-grade systemic inflammation contributing to metabolic dysfunction (reviewed in [48]). Clinically, type 2 diabetes is evidenced by a rise in blood glucose due to faulty insulin-dependent signaling mechanisms. Pio-dependent glucose normalization did not require weight loss in the BBZDR/Wor type 2 diabetes rat model (Fig. 1a, b), suggesting that PPARγ regulates additional mechanisms besides adipose tissue and body weight. The increase in adipose tissue observed in type 2 diabetes has led to focus investigations of the pro-inflammatory mediators associated with visceral adipose tissue (VAT). Two major cell populations have been investigated in the past, VAT regulatory T cells [49–53] and macrophages [23, 35, 48, 54–56]. First, Cipolleta et al., recently showed regulatory T cells expressing PPARγ suppress inflammation and regulates insulin resistance [49, 50]. Clark and colleagues [57] demonstrated Ciglitazone, another PPARγ agonist, enhances regulatory T cells in vitro. Accordingly, our work shows upregulation of CD25 in Pio-treated splenocytes in vitro. A large portion of the CD25^+^ cells were positive for the FoxP3 transcription factor, considered a master regulator of natural regulatory T cells [40–42]. We and others, have previously shown enhancement of the FOXP3 transcription factor in human cells correlates with increases in suppressive capacity of regulatory cells [58, 59]. Thus we speculate this population makes a major contribution to dampening of inflammation. We also speculate that the remaining FoxP3^neg^ population exhibits an activated or induced regulatory T cell phenotype.

A number of reports have established that monocytes recruited to adipose tissue become resident macrophages [60, 61]. Macrophages accumulate in adipose tissue in both obese patients and rodents [35]. This prompted the studies to establish the linkage between an inflammatory phenotype characterized by increased levels of TNF-α and other pro-inflammatory cytokines and insulin resistance. Resident macrophages in adipose tissue of lean rodents showed an anti-inflammatory phenotype, or M2, whereas those of obese rodents showed a pro-inflammatory, or M1, phenotype. M1 macrophages displayed high levels of CD11c in their surface. While the mechanisms responsible for recruitment and establishment of subtypes of macrophages in VAT in diabetes are beginning to be understood, there is relatively little understanding of comparable mechanisms in other immune organs. Similar to the VAT findings, we observed a higher percentage of CD11b/c^+^ cells in splenocytes from diabetic rats compared to non-diabetics (Fig. 3a). This population decreased in the Pio-treated rats. Moreover, the population was not altered in the Pio-treated non-diabetic rats. These results suggests that diabetes-dependent inflammation causes an increase in the CD11b/c^+^ in splenocytes and that Pio-treatment reduces this population to baseline levels, as evidenced by the lack of effects in the untreated and treated non-diabetic rats.

Much of the pro-inflammatory cytokine milieu is attributed to the M1 population in VAT [39, 54, 62]. Our work revealed that non-VAT components are also contributors to the inflammation. We found CD3^+^ T lymphocytes in diabetic rats produced higher levels of TNF-α and IL-1β, both pro-inflammatory mediators, compared to non-diabetic counterparts (Fig. 2a, b). Both pro-inflamatory cytokines were significantly reduced in splenocytes of Pio-treated rats, suggesting that Pio suppresses cell immune responses in non-adipose tissue. Of particular note is the reduction in IL-1β, as this cytokine is implicated in the destruction of pancreatic β-cells [63, 64]. Reduction of pro-inflammatory cytokines may not be sufficient to control the disease. This may also be accompanied by an increase in anti-inflammatory cytokines, such as IL-4. Classically, IL-4 serves as an immunoregulatory cytokine and as a mitogen for the proliferation of cells with an anti-inflammatory phenotype [65–67]. It is important to determine the source of the IL-4 production. Our work revealed that in the spleen, CD3^+^ T lymphocytes, not CD3^neg^ cells, were a major source of IL-4 (Fig. 2c). Together, this work suggests splenic components are contributors to the control of inflammation in Pio-treated diabetic rats.

Using our BBZDR/Wor rat model, we found Pio treatment in vivo caused an increase in recent thymic emigrants, which may contribute to the increase in percentage of total T lymphocytes, resulting in alleviation of lymphopenia observed in untreated BBZDR/Wor rats. Previous work in type 1, not type 2, diabetes rat models showed reduction of recent thymic emigrants is due, in part to defective intrathymic selection leading to autoimmunity [68]. Subsequent reports by Ramanathan and colleagues [69] confirmed these results. Rodent models of obesity based on mutations in leptin-coding gene (*Lep*^*ob*^*/Lep*^*ob*^) have shown thymic involution that reduces T cell responses and the number of T cell emigrants to the periphery [70]. This suggests that there is a compound effect of diabetes coupled with obesity. Here, we show first, type 2 diabetic BBZDR/Wor rats have reduced frequency of recent thymic emigrants and second, a Pio-dependent increase in recent thymic emigrants in diabetic rats, independent of their body weight. Our work suggests the effects are Pio-dependent as we observed an increase in recent thymic emigrants in non diabetic rats with normal blood glucose levels. Still, we cannot rule out there might be compound effects by the reduction in blood glucose in addition to Pio-treatment, as shown in the diabetic + Pio group. Recent work suggests Pio and other TZDs can act in PPARγ dependent and independent manner (reviewed in [71]). This new data provides an additional complexity level to our study and others as it demonstrated reduction in the pro-inflammatory milieu could happen independent of PPARγ signaling in the presence of Pio and other TZDs. Our work shows the presence of PPARγ in the rats hepatocytes and splenocytes. Interestingly, our study opens new investigations to integrate multiple signaling pathways that could facilitate type 2 diabetes therapeutics. Some provocative scientific concerns are if other blood glucose lowering drugs will exhibit similar in vivo results; and how does insulin receptor signaling is affected by Pio. There is a need for more biochemical studies to understand the basis of Pio’s actions.

Our investigation suggests there is promising potential for immune modulation therapy in type 2 diabetes using the TZD-derivative, Pioglitazone. More work is required to elucidate how the PPARγ-signaling pathway, via Pio, interacts with multiple cellular pathways, including surface receptors, signals driving proliferative and suppressive responses, and transcriptional regulators. Future studies aimed at investigating how to improve immune surveillance and control the complications of type 2 diabetes could provide new strategies for improving the quality of life of diabetic patients.
